# Supplementary material for: Validation of Enzyme Immunoassays via an Adrenocorticotrophic Stimulation Test for the Non-Invasive Quantification of Stress-Related Hormone Metabolites in Naked Mole-Rats
Source: Animals (Basel). 2023 Apr 21;13(8):1424. doi: 10.3390/ani13081424 (PMC10135013; doi:10.3390/ani13081424)
Supplement: Supplementary file 1 [file animals-13-01424-s001.zip › animals-2329239-supplementary.pdf]

Supplementary Materials:

Table S1. Summary of saline volume and synthetic adrenocorticotropic hormone (Synacthen® depot, Novartis, South Africa (Pty) Ltd.) administered to each individual during the ACTH challenge.

ID	Sex	Dosage (μg/100g)	Mass (g)	Synacthen Volume (μL)	Saline Volume (μL)
F1	Female	20	45.1	36	164
F2	Female	20	35.6	28	172
F3	Female	20	38.2	31	169
M1	Male	20	53.3	43	157
M2	Male	20	40.5	33	167
M3	Male	20	38.5	31	169
F4	Female	60	43.0	103	97
F5	Female	60	56.4	135	65
F6	Female	60	50.1	120	80
M4	Male	60	35.5	85	115
M5	Male	60	33.2	80	120
M6	Male	60	39.4	95	105

Table S2. Summary of EIA sensitivities and, intra-assay and inter-assay coefficients of variation of high and low value quality controls for all EIAs applied to measure immunoreactive urine glucocorticoid metabolite (uGCM) and fecal glucocorticoid metabolite in naked mole-rats.

Enzyme immunoassay	Sensitivity		Intra-assay variation (%)	Inter-assay variation (%)
	**Urine (pg/mL)**	**Feces (ng/g fecal dry mass)**		
Corticosterone	80	2.4	4.57–5.74	10.60–13.49
Cortisol	25	0.75	4.42–6.24	14.11–14.89
11-oxoaetiocholanolone I	40	1.2	4.09–4.81	14.28–14.85
11-oxoaetiocholanolone II	25	0.75	4.94–6.84	10.91–12.74
5α-pregnane-3β,11β,21-triol-20-one	80	2.4	4.71–5.71	9.98–12.81
